# High number of refugees in Germany - how is the mental health care dealing with this major challenge?

**DOI:** 10.1192/j.eurpsy.2023.177

**Published:** 2023-07-19

**Authors:** M. Schouler-Ocak

**Affiliations:** Psychiatric University Clinic of Charité at St. Hedwig Hospital, Berlin, Germany

## Abstract

**Abstract:**

Europe is again confronted with a new dramatic emergency, a war which has already caused civil victims, mass displacement and even fear about a nuclear war and energy crisis. Again, Europe is facing new waves of war refugees, forcibly displaced people. There is increasing evidence that a large proportion of refugees or forcibly displaced persons suffer from the consequences of traumatic events and exhibit psychological problems or develop mental disorders, including post-traumatic stress disorder, depressive and anxiety disorders, and relapses in psychotic episodes. European countries are trying to face with an extraordinary surge of solidarity and generosity, but at the same time with the awareness that the needs are much beyond reaction capacity of individual people and states. The direct and indirect consequences of this humanitarian catastrophe cannot be estimated at present. Mental healthcare services are suddenly faced with major challenges and need to develop or expand strategies to address them. In this presentation, strategies from Germany will be presented and discussed.

**Disclosure of Interest:**

None Declared

